# Impact of polyethylene terephthalate nanoplastics (PET) on fibroblasts: a study on NIH-3T3 cells

**DOI:** 10.3389/fphys.2025.1580682

**Published:** 2025-06-09

**Authors:** Maria Elena Giordano, Francesca Lionetto, Maria Giulia Lionetto

**Affiliations:** ^1^ Department of Biological and Environmental Sciences and Technologies, University of Salento, Lecce, Italy; ^2^ Department of Engineering for Innovation, University of Salento, Lecce, Italy; ^3^ NBFC, National Biodiversity Future Center, Palermo, Italy

**Keywords:** wound healing assay, cellular internalization, oxidative stress, cell migration, confocal microscopy, nanoplastics, autofluorescence, hansen solubility parameters

## Abstract

Plastic pollution has become a major environmental and public health issue due to rising global production. Nanoplastics (NPs) are especially concerning due to their widespread presence and potential health risks. This study aims to determine the impact of the exposure to polyethylene terephthalate (PET) NPs on fibroblast cells using the murine NIH-3T3 cells as experimental model. This is a relevant cellular model for several biological fields of application, including cell migration in wound healing and tissue regeneration. The PET NPs used represented an environmentally realistic PET NPs model since they were produced by a fast top down approach in a process close to the mechanical abrasion of microplastics occurring in the environment. They were characterized by an intrinsic autofluorescence which enables their use in studies of NPs interactions with biological systems without the need for additional fluorescent dyes. Additionally, the Hansen solubility parameters (HSP) of the PET NPs and the culture medium were determined to better understand their interaction. PET NPs were internalized by fibroblasts in a dose-dependent manner, localizing in the cytoplasm. While they caused only a slight reduction in cell viability (within 20% inhibition at 10–100 μg/mL) after 24 h exposure, they significantly impaired fibroblast migration, as demonstrated by the scratch assay, indicating possible interference in tissue repair. The exposure of the cells to PET NPs induced a significant dose-dependent ROS increase suggesting the induction of intracellular oxidative stress as possible mechanisms underlying the observed migration impairment. These findings highlight the potential risks of PET NPs to fibroblasts, emphasizing the need for further research into their impact on cellular functions and mechanisms.

## 1 Introduction

In recent years plastic pollution has emerged as a critical environmental and public health problem due to plastic production reaching unprecedented levels all over the world (Bidashimwa et al., 2023). Particularly, nanoplastics (NPs)—plastic particles smaller than 1 μm (Gigault et al., 2018), that can arise from the breakdown of larger plastics or be purposefully manufactured, have gained attention due to their ubiquity across various environmental matrices, raising significant health concerns. Recently, the presence of micro and nanoplastics (MNPs) in human kidney, liver and brain has been confirmed rising concern about bioaccumulation processes (Nihart et al., 2025). MNPs can enter the body through several pathways, including ingestion, inhalation, and dermal contact (Zarus et al., 2021). They have been detected in various food items and drinking water sources (Khan and Jia, 2023). MNPs can originate from various sources, including synthetic fibers and urban dust (Dube and Okuthe, 2023). Dermal contact with MNPs occurs through the use of personal care products containing microbeads, contact with contaminated water, and exposure to airborne MNPs present in the atmosphere that can settle with dust and come into contact with the skin (Abafe et al., 2023). Therefore, the skin may be an important route of MNPs entry into the body (Celebi Sözener et al., 2020). While the skin acts as a barrier, certain conditions may facilitate the penetration of nanoparticles such as skin damage (Larese Filon et al., 2016).

The research on the biological and health related effects of NPs has made significant progress in the last years (Swee-Li Yee et al., 2021) due to the worldwide distribution of this emerging pollutant whose contamination involves all environmental matrices. Particularly, the studies at the cellular level contributed to understanding the molecular and cellular mechanisms underlying the health impact of this emerging pollutants. While there is a growing body of literature examining the impacts of MNPs on various cell types, detailed investigations into their effects on fibroblasts are limited. Fibroblasts play a crucial role in tissue repair, wound healing, and maintaining the extracellular matrix (Plikus et al., 2021). They produce collagen, providing tensile strength and resistance to mechanical stress in tissues (Tracy et al., 2016), remodel the extracellular matrix in response to mechanical signals and its synthesis and degradation (Kendall et al., 2014). Upon injury, fibroblasts become activated and migrate to the wound site, contributing to tissue repair (Kendall et al., 2014). Fibroblasts release cytokines and chemokines, recruiting immune cells and modulating inflammation (Silzle et al., 2003). They also produce Vascular Endothelial Growth Factor (VEGF) and Fibroblast Growth Factors (FGF), promoting endothelial proliferation and blood vessel formation (Ollivier et al., 2000).

The few works available in the literature on the effect of MNPs on fibroblasts have demonstrated that these cells can internalize polystyrene NPs, leading to dose-dependent adverse effects on cellular processes (Peng et al., 2024). These effects vary across different fibroblast cell lines, emphasizing the need for further exploration to understand the implications for human health (Peng et al., 2024). Exposure to polystyrene NPs has been shown to induce significant changes in gene expression and DNA methylation patterns in human dermal fibroblasts, altering the normal cellular functions (Stojkovic et al., 2023). Moreover, aminated polystyrene nanoparticles exhibit dose- and size-dependent cytotoxicity in HFF-2 fibroblasts, with smaller sizes and higher concentrations increasing oxidative stress, apoptosis, and cell cycle arrest (Sadeghinia et al., 2025). Most of the data to date available on the effect of MNPs on fibroblasts refers to polystyrene NPs. Only one study evaluated the impact of environmentally more realistic microplastics of different sizes, sourced from various depths of the Adriatic Sea, on human gingival fibroblasts (hGFs). These microplastics were found to induce inflammatory responses, suggesting that environmental microplastics can affect fibroblast function (Caputi et al., 2022). These results outline the need to expand the research of the effect of MNPs at the cellular levels to other types of plastics materials that can be found in the environment. Due to their extensive production, use, and mismanagement, different micro- and nanosized thermoplastic polymers can be found in the environment, such as polyethylene (PE), polyethylene terephthalate (PET), and polypropylene (PP) (Lionetto et al., 2023).

Research on the cellular effects of polyethylene terephthalate (PET) NPs is currently less extensive compared to studies on other types of NPs, such as polystyrene. While there is a growing body of literature examining the impacts of various NPs on cellular functions, PET-specific studies remain limited. PET is one of the most widely used plastics, found in bottles, textiles, and food packaging (Lionetto et al., 2021; Lionetto et al., 2022). It is a major source of environmental MNPs due to its widespread use and improper disposal. PET degrades slowly, leading to long-term accumulation in ecosystems and increased human exposure through water, food, and air (Lionetto et al., 2021). Therefore, understanding the cellular effects of PET particles is critical due to their widespread presence and persistent nature in the environment.

This study aims to advance understanding of the effects of PET NPs on murine NIH-3T3 fibroblasts, with a focus on cell migration and intracellular redox balance. NIH-3T3 cells represent a versatile fibroblast model widely used in molecular biology, biomedical research, pharmacology, and toxicology (Rahimi et al., 2022), and are particularly suitable for studying migration processes essential to wound healing and tissue regeneration (Giannakopoulos et al., 2023). The PET NPs used in this study were obtained by a fragmentation process close to the mechanical abrasion of microplastics occurring in the environment, according to Lionetto et al. (2022). They were characterized by an intrinsic fluorescence making them useful in the study of NP interaction with biological systems (Lionetto et al., 2022).

## 2 Materials and methods

### 2.1 PET nanoplastic production and characterization

Model PET nanoparticles were generated following the method presented in our earlier research (Lionetto et al., 2022), using RT52 PET pellets provided by Invista Resins & Fibers GmbH (Gersthofen, Germany) (Lionetto et al., 2021). This approach employed a top-down mechanical fragmentation technique, which mimicked the natural abrasion of microplastics by sand granules in aquatic environments. The method does not require the use of solvents.

Dynamic light scattering (DLS) measurements were carried out at room temperature using a Malvern Zetamaster Nano-ZS. The measurements were carried out on the diluted suspensions, suitable for light-scattering measurements.

FT-IR analyses allowed the identification of the functional groups present on PET polymer. Fourier transform infrared spectroscopy (FT-IR) was performed with a Jasco 6300 FT-IR 203 spectrometer (JASCO Corporation, Tokyo, Japan). Infrared spectra were recorded in the wavelength range between 750 cm^-1^ and 3,500 cm^-1^ with 128 scans and 4 cm^-1^ of resolution, by using ATR Pro One X with ZnSe crystal.

The autofluorescence of PET NPs was spectrofluorimetrically characterized in our earlier research (Lionetto et al., 2022). In this work the autofluorescence of PET NPs was assessed by confocal microscopy using a 405 nm laser line of A1 NIKON confocal laser scanning unit (emission filter 425–475 nm) coupled with a NIKON Ti microscope.

### 2.2 Cell culture and exposure to PET NPs

The effects of PET NPs were assessed on NIH-3T3 fibroblast cell line (ATCC^®^ CRL-1658™) derived from mouse embryos. These cells are widely used in various fields of cell biology research and represent an established fibroblast model for assessing cellular responses to external agents (Boncler et al., 2018; Wagner et al., 2022).

NIH-3T3 cells were cultured in Dulbecco’s modified Eagle medium (D-MEM) (EuroClone Paignton-Devon, United Kingdom) supplemented with 10% (v/v) fetal bovine serum, 2 mM L-glutamine, and 100 μg/mL penicillin/streptomycin, in a humidified atmosphere (5% CO_2_ in air) at 37°C. For experimental exposure to PET NPs, cells (concentrated 2 × 10^4^ per ml) were seeded in a 96-well plate for 24 h. Thereafter, they were incubated with PET NPs for 24 h at different concentrations (0, 10, 25, 50, and 100 μg/mL). Before incubation with the cells, the dispersion of PET NPs in D-MEM medium was sonicated with a customized protocol using a Bioruptor Plus (Diagenode, Denville, NJ, United States) in order to keep a well dispersed state for the duration of the exposure experiment.

All experiments were performed between passages 3 and 10 of propagation. Every exposure was performed in three replicates.

The selected concentration range (10–100 μg/mL) used in this study is included in the concentrations normally used in *in vitro* studies with PET NPs (Gettings et al., 2024; Aguilar-Guzmán et al., 2022; Zhang et al., 2022; Magrì et al. 2018) to facilitates comparison across different studies and experimental setups in evaluating the biological impacts of PET. A recent study by Leslie et al. (2022)) detected MNPs in human blood at concentrations ranging from 1 to 7 μg/mL, with PET identified as one of the most prevalent plastic components. The range of concentrations used in our study is one order of magnitude higher than the concentration range measured in human blood. This discrepancy is justified by the intrinsic characteristics of *in vitro* studies, where higher concentrations are commonly employed to induce detectable biological effects within short exposure periods. Furthermore, *in vitro* experimental exposure is designed to simulate, over limited timescales, exposure conditions that *in vivo* would occur over much longer durations, potentially leading to accumulation phenomena over time.

### 2.3 Confocal visualization on living cells

NIH-3T3 cells (concentrated 2 × 10^4^ per ml) were seeded in a tissue culture-treated µ-Slide 4 well (Ibidi GmbH, Gräfelfing, Germany) for 24 h and then they were incubated with PET NPs for 24 h. After incubation cells were washed three times to remove NPs and were visualized by a 405 nm laser line of A1 NIKON confocal laser scanning unit using coupled with a NIKON Ti microscope. Cells were visualized by a Plan Apo 60×1.40 Oil objective (Nikon, Tokyo, Japan).

### 2.4 Cell viability assessment by MTT test

The effect of PET NP exposure on NIH-3T3 cell viability was assessed using MTT (3-(4,5-dimethylthiazol-2-yl)-2,5-diphenyltetrazolium bromide) test (Sylvester, 2011; Rajabimashhadi et al., 2024). MTT assay is one of the most widely used and well-established methods for assessing cell viability *in vitro*. It is frequently applied in several field of cell research and it is the most prevalent method for assessing cell viability in studies of MNPs (Ferreira et al., 2024). In this regard, the use of the MTT assay in this work allows comparing the cell viability results obtained following exposure to PET NPs with results obtained on other cell types and other NP types.

The test evaluated the mitochondrial NAD(*P*)H-dependent oxidoreductase enzyme activity that can reduce a yellow tetrazolium salt (MTT) to a purple formazan. This in turn accumulated as crystals within healthy cells. The crystals were in turn dissolved with DMSO and the absorbance of the resulting coloured solution was spectrophotometrically analyzed at 570 nm (Cytation 5, BioTek Instruments, Winooski, VT, United States). The relative viability of the cells was calculated by Equation 1:

$$\%\text{Relative viability of cells}=\frac{\text{Treated}\;\text{cells}\;\text{OD}}{\text{Control}\;\text{cells}\;\text{OD}}*100$$ (1)

### 2.5 Wound healing and cell migration assay

The spreading and migration capabilities of 3T3 fibroblasts exposed to PET NPs were measured using a scratch wound assay. First, NIH 3T3 cells were seeded in six-well plates at a density of 5 × 10^5^ cells/well in DMEM, then the cells were incubated at 37°C and 5% CO_2_ until they reached a confluence of approximately 80%. Then, an *in vitro* wound model was established by producing a linear scratch across the fibroblast cell layer of the six-well culture plate using a sterile 200 μL pipette tip in a single motion to simulate a wound. Any cellular debris was removed by replacing the medium. Then the cells were exposed to PET NPs 50 μg/mL for 24 h. Images were taken at the same location of each well before and after 24 h by Cytation 5 multimode reader. The wound closure rate was measured using image analysis software ImageJ after downloading the plugin Wound Healing Size Tool (Suarez-Arnedo et al., 2020), that automatically recognizes the size of the scratch wound, corrects the average wound width considering its inclination and quantifies parameters such as area, fraction of wound area, average wound width and width deviation of the wound images.

The wound closure percentage was assessed according to Grada et al. (2017) using Equation 2:

$$\text{Woundclosure}\%=\frac{A_{t=0}-{A_{\Delta }}_{t}}{A_{t=0}}*100$$ (2)

### 2.6 Intracellular oxidative stress detection

The intracellular oxidative stress was evaluated using the cell-permeant probe sensitive to intracellular reactive species 5-(and-6-)-chloromethyl-2,7-dichlorodihydrofluorescein diacetate acetyl ester (CM-H_2_DCFDA) (Ex/Em: 492–495/517–527 nm) (Thermo Fisher Scientific, Waltham, MA, United States). It is a commonly used probe for detecting Reactive Oxygen Species (ROS) formation in cells (King and Oh, 2004; Giordano et al., 2023; Giordano and Lionetto, 2023; Giordano et al., 2020a; Giordano et al., 2020b). Once inside the cell, intracellular esterases cleave the acetate groups of CM-H_2_DCFDA, converting it into DCFH, which remains entrapped within the cell. Oxidation of DCFH by intracellular oxidants (via a two-electron process) produces the fluorescent product DCF (Forman et al., 2015). Cells were plated into Corning™ 96-well black/clear bottom plate TC surface for 24 h to allow the cell attachment. Then, the cells were incubated for 24 h with PET NPs in a range of concentrations (0, 10, 25, and 50 μg/mL). Then, they were washed three times to remove residual PET NPs and were charged with CM-H_2_DCFDA according to (Giordano et al., 2020a; Giordano et al., 2020b). Briefly the cells were incubated with 5 µM CM-H_2_DCFDA for 30 min at 37°C and then washed to remove the extracellular dye. Fluorescence was then measured by Cytation 5™ (BioTek Instruments, Inc., Winooski, VT, United States) multi-mode microplate reader. The results are expressed as percentage variation of the fluorescence intensity with respect to the control and were calculated as follows:

$$\text{Percentage Variation}=\left(\left({\text{FI}}_{\text{sample}}-{\text{FI}}_{\text{control}}\right)/\;{\text{FI}}_{\text{control}}\right)\times 100$$

where FI_sample_ is the fluorescence intensity of the test sample, FI_control_ is Fluorescence intensity of the control sample.

In order to assess the intracellular localization of the de-esterified fluorescent probe, the cells charged with CM-H_2_DCFDA were also visualized by fluorescent microscopy using the multi-mode microplate reader Cytation 5 using a 40x objective.

### 2.7 Statistical analysis

All the experiments were performed in triplicate. Statistical tests utilized to evaluate the statistical significance of differences were Student t test, One Way ANOVA, and Dunnett’s post-test as indicated in the figures’ captions. Data are expressed as mean ± SEM.

## 3 Results

### 3.1 PET NPs characterization

**FIGURE 1:**
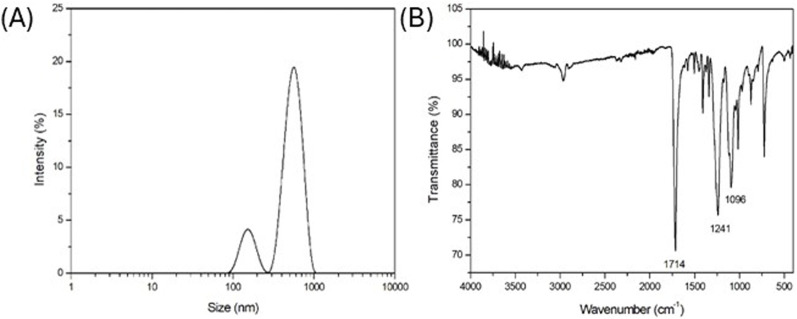
**(A)** DLS intensity-based size distribution (representative of n = 3 independent experiments); **(B)** FT-IR spectrum (representative of n = 3 independent experiments) of laboratory made PET NPs.

As reported in Figure 1A where the DLS intensity-based size distribution is shown (representative of n = 3 independent experiments), the used PET nanoparticles exhibited a bimodal size distribution, with two distinct peaks, suggesting the presence of two populations of particles with different sizes centered at 150 nm and 570 nm. In a previous work of the authors, it was demonstrated that the obtained PET NPs exhibited irregular shapes and surfaces, reflecting characteristics of environmentally occurring secondary NPs formed from the degradation of larger plastic items (Lionetto et al., 2022).

FT-IR spectrum in Figure 1B (representative of n = 3 independent experiments) highlighted the key functional groups of PET. In particular, a strong and prominent peak at 1714 cm^-1^, typical indicator of the ester carbonyl (C=O) stretching vibration, was observed. Additionally, the peaks at 1,241 cm^-1^ and 1,096 cm^-1^ were related to the C-O stretching vibration in the ester group, due to the aliphatic and aromatic ester, respectively, being a significant feature for identifying ester linkages. The FT-IR spectrum confirmed the preservation of molecular structure of PET NPs without any chemical modification due to the production process.

To better understand the interaction between Dulbecco’s modified Eagle medium (D-MEM) supplemented with antibiotic and PET NPs, the Hansen solubility parameters (HSPs) were calculated. HSPs are a set of three parameters, named dispersion or non-polar interaction (δ_D_), polar or dipole-dipole interaction (δ_P_), and hydrogen bonding interaction (δ_H_), as given by Equation 3 (Hansen, 2007):

$$\delta_{T}^{2}=\delta_{d}^{2}+\delta_{p}^{2}+\delta_{h}^{2}$$ (3)

These parameters quantify the cohesive energy of a material and predict solubility behavior based on the “like dissolves like” principle. HSPs of PET NPs and D-MEM solution were estimated by the group contribution method by summing the contributions of individual structural groups within a molecule (Greco et al., 2015). This method allows for rapid estimation when experimental data is unavailable. Since D-MEM cell culture medium is a multicomponent solution supplemented with antibiotics, HSPs of the mixture have been calculated using a weighted average of the individual component parameters, i.e., water, salts, sugars, amino acids, vitamins and antibiotic (Hansen, 2007; Yadav et al., 2023; Lionetto et al., 2023) based on their volume fractions Φ_i_, as follows (Equation 4):

$$\delta_{\text{mix}}=\sum \left(\Phi_{i}*\delta_{i}\right)$$ (4)

The obtained Hansen solubility parameters for PET nanoparticles and D-MEM medium are reported in Table 1.

**TABLE 1:** Hansen solubility parameters.

Material	δ_d_ (MPa)^0.5^	δ_p_ (MPa)^0.5^	δ_h_ (MPa)^0.5^	δ_T_ (MPa)^0.5^
PET	18.2	6.4	6.6	20.4
D-MEM medium	15.5	16.0	42.2	47.7

**FIGURE 2:**
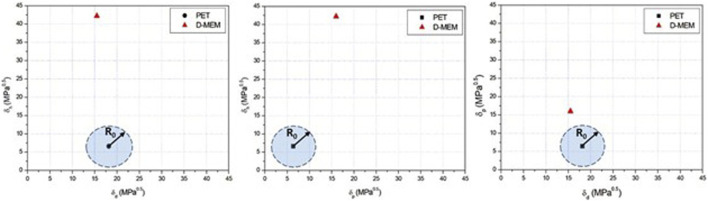
Hansen solubility parameter 2D plots for PET and D-MEM medium (representative of n = 3 independent experiments).

With the HSPs reported in Table 1, a solubility sphere centered at PET HSPs can be drawn with an interaction radius R_0_ equal to 5 (MPa)^0.5^ for PET polymer [1], that defines the limit of good solubility or compatibility. To simplify visualization, the 3D sphere is projected as a blue circle area onto three 2D planes in Figure 2, to help assess how close the culture medium lies to the solubility sphere. Since D-MEM falls outside the R_0_ radius of PET HSP circle area in all planes (Figure 2), it is energetically unfavorable for it to interact closely with the polymer. This implies that D-MEM medium does not penetrate the PET matrix which cannot swell since swelling of nanoparticles typically requires some degree of solubility.

**FIGURE 3:**
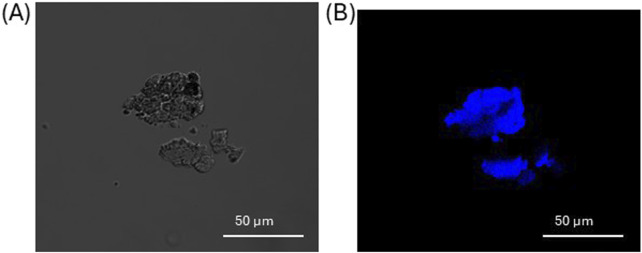
Aggregates of PET NPs powder visualized in brightfield **(A)** and confocal microscopy **(B)** by a 405 nm laser line of A1 NIKON confocal laser scanning unit coupled with a NIKON Ti microscope. Objective used 20X. Images representative of n = 3 independent experiments.

The laboratory made PET NPs possessed inherent autofluorescence (Lionetto et al., 2022). When excited at 405 nm they expressed a maximum emission at 450 nm. As shown in Figures 3A,B, which presents a representative image of PET NP powder aggregates captured in brightfield (A) and confocal microscopy (B) (from n = 3 independent experiments), PET NPs were clearly visible using confocal microscopy with a 405 nm excitation laser line and a 425–475 nm emission filter. This laser line is commonly used in biological research to excite fluorophores with excitation peaks in the near-UV to violet range. This property enables the use of these PET NPs in studies of NPs interactions with biological systems in any fluorescence procedure without the need for additional fluorescent dyes. Moreover, the solvent-free production of these PET NPs offers a key advantage for biological sample applications.

### 3.2 PET NPs internalization

**FIGURE 4:**
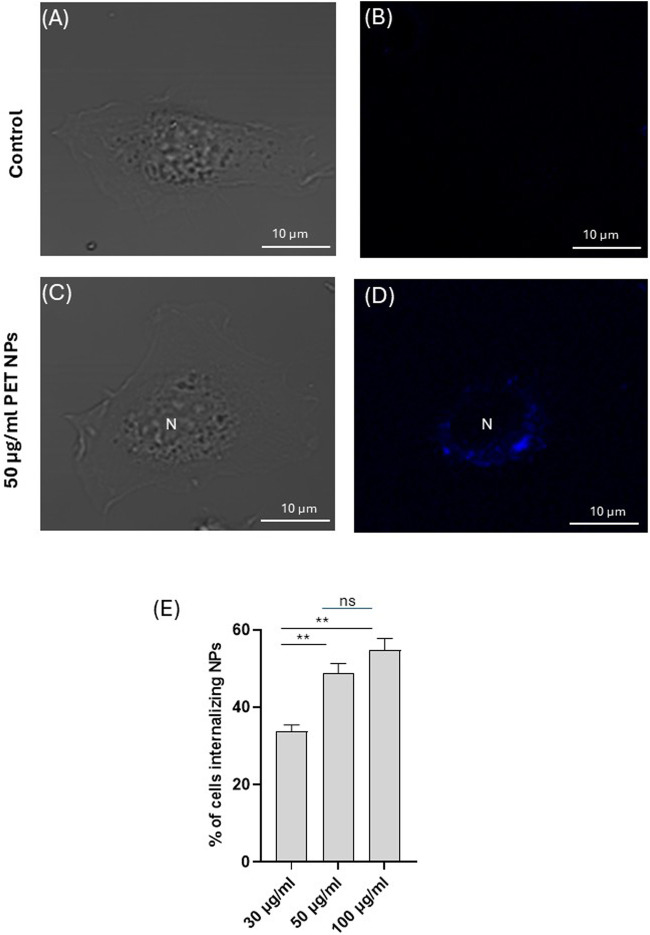
Representative image (of n = 3 independent experiments) of a 3T3 fibroblast exposed to PET NPs (50 μg/mL) for 24 h visualized in brightfield **(C)** and confocal microscopy **(D)** by a 405 nm laser line of A1 NIKON confocal laser scanning unit coupled with a NIKON Ti microscope. A control cell **(A,B)** is shown for comparison. Objective used 60X oil immersion. **(E)** % of cells internalizing PET NPs at different NP concentrations (data are expressed as mean ± SEM of three independent experiment).

When 3T3 cells were exposed to PET NPs for 24 h, they showed a cytoplasmatic localization as assessed by confocal microscopy observation on living cells (Figures 4A–E; Supplementary Figure S1, representative images of n = 3 independent experiments), suggesting the ability of PET NPs to be internalized into the cells. The NP internalization appeared dose-dependent in the range of concentrations tested (from 30 μg/mL to 100 μg/mL) (Figure 4E). In particular, at the highest concentration tested we did not observe a proportional increase in the percentage of cells internalizing NPs.

### 3.3 Cytotoxicity

**FIGURE 5:**
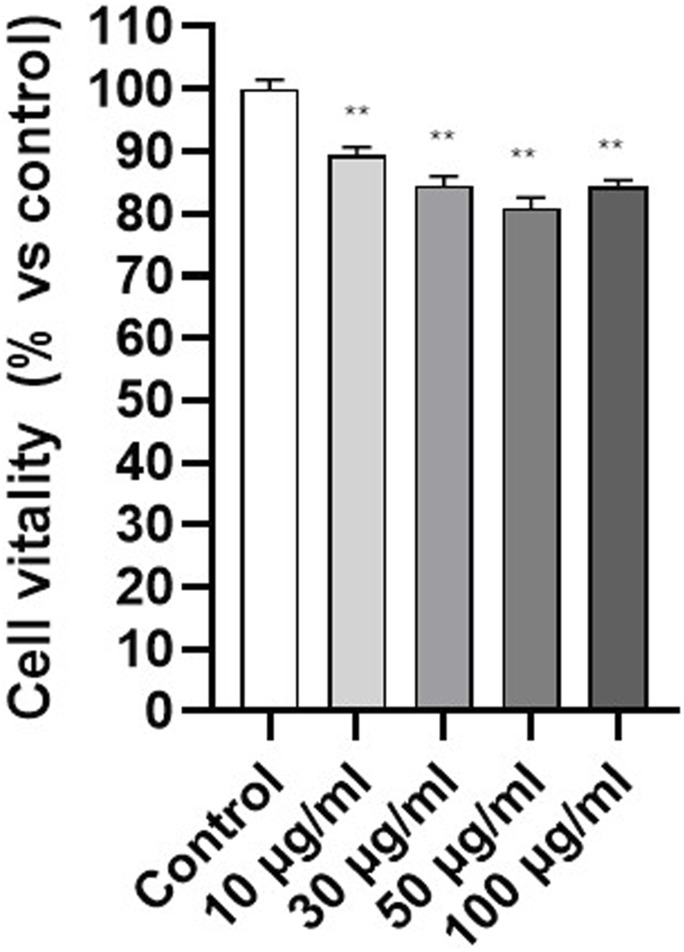
Effect of PET NPs exposure (24 h) on 3T3 cell vitality assessed by the MTT test. **P < 0.01 (One Way ANOVA and Dunnett post-test). Data are expressed as mean ± SEM of three independent experiments.

After demonstrating that PET NPs can be internalized by fibroblasts, we investigated their cellular effects starting with MTT test on 3T3 cells exposed to PET NPs for 24 h (concentration range from 10 μg/mL to 100 μg/mL) for the assessment of potential effects on cell viability. Data are expressed as mean ± SEM of three independent experiments. A slightly dose-dependent decline in cell viability was observed (Figure 5). It reached a significant reduction of about 20% at the PET NP concentration of 50 μg/mL. This finding indicates a potential slight cytotoxic effect of PET NPs on NIH-3T3 fibroblasts by interfering with the cellular metabolism. The further increase of the concentration to 100 μg/mL did not produce further increase in the viability reduction. MTT assay measures cellular metabolic activity as an indicator of cell viability, but it may not fully capture other forms of cell stress. A plateau might indicate that metabolic impairment has reached a detectable limit, even if other toxic effects are still occurring.

### 3.4 Wound healing and cell migration

Fibroblasts are known for their roles in wound healing and cell migration, being essential in the repair and regeneration of tissues following injury (Plikus et al., 2021). In order to assess if any functional interferences in cell migration or mobility were induced in the cells following PET NPs exposure, the scratch wound assay was performed on cells exposed for 24 h to 50 μg/mL PET NPs, the concentration that resulted in the maximum effect of a 20% reduction in vitality in the MTT assay.

**FIGURE 6:**
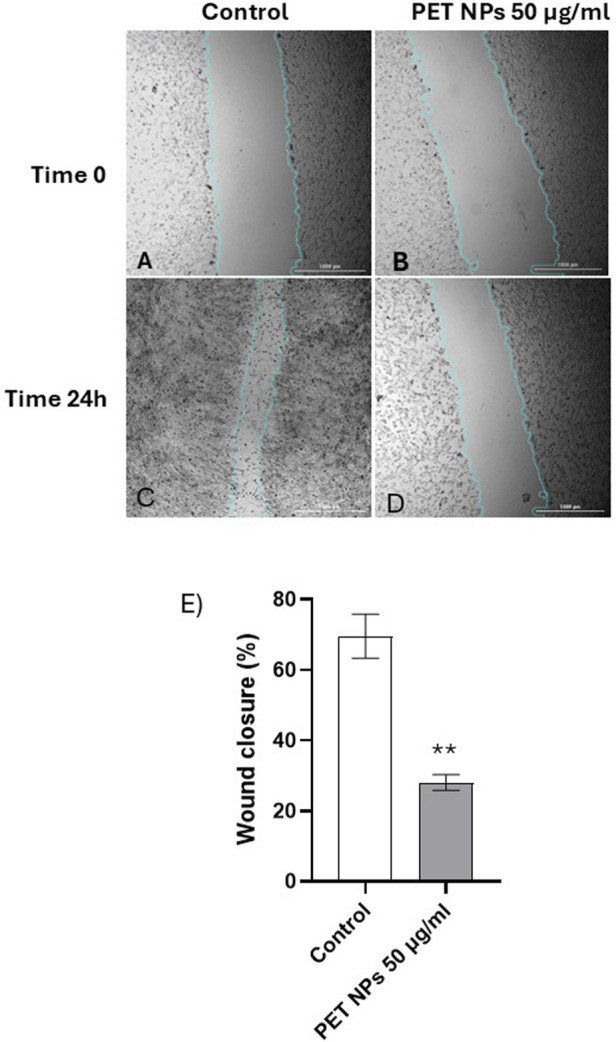
**(A–E)**. Representative bright-field images of scratch test on NIH 3T3 cell acquired by the imaging multimode reader Cytation 5 Biotek (obj 4x). **(A)** Control group (t:0 h), **(C)** Control group (t:24 h), **(B)** cells exposed to NPs 100 μg/mL (t:0 h), **(D)** cells exposed to NPs 100 μg/mL (t:24 h). The light blue line was automatically applied by the image analysis software used (see Methods) for recognizing the size of the scratch wound and measure wound closure. **(E)** Wound closure percentage calculated in control and PET NPs (50 μg/mL) exposed cells for 24 h **P < 0.01 (Student t test). Data are expressed as mean ± SEM of three independent experiments.

As shown in representative Figures 6A–D the exposure of the cells to PET NPs significantly inhibited the migration of the cells and in turn wound closure after scratch. The quantification of the effect (Figure 6E) corresponded to about 60% reduction in the wound closure percentage.

### 3.5 Oxidative stress

**FIGURE 7:**
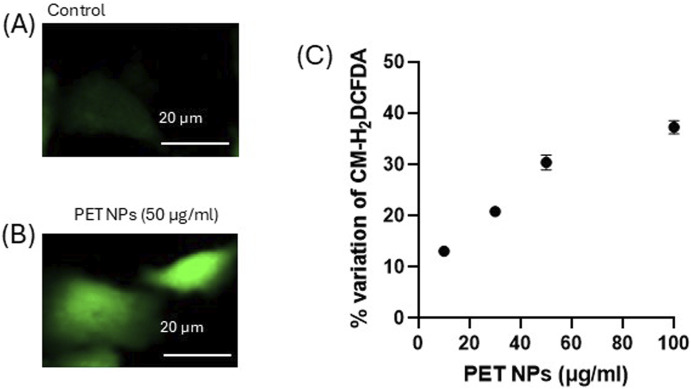
**(A,B)** Representative fluorescence microscopy images (of n = 3 independent experiments) of control and PET NP exposed (24 h) 3T3 cells charged with the ROS sensitive probe CM-H_2_DCFDA for the intracellular ROS assessment; **(C)** % variation of the fluorescence intensity of 3T3 cells exposed for 24 h to increasing concentrations of PET NPs and then charged with CM-H_2_DCFDA. **P < 0.01 (One Way ANOVA and Dunnett post-test). Data are expressed as mean ± SEM of three independent experiments.

Various studies have shown that NPs can activate oxidative stress pathways, leading to alterations in cellular functions (Kaluç et al., 2024; Wu et al., 2024; Ferrante et al., 2022). Moreover, intracellular reactive oxygen species (ROS) are considered key regulators of cell motility (Huang et al., 2013). To study the mechanisms underlying PET NP induced effects on 3T3 cells, the possible induction of oxidative stress was investigated. The cells where exposed to different concentrations of PET NPs for 24 h and then charged with the cell-permeant ROS sensitive probe CM-H_*2*_DCFDA. As observed by fluorescence microscopy and quantified by spectrofluorimetry (Figure 7), the exposure of the cells induced a significant increase of the fluorescence of the probe compared to control (expressed as percentage variation of the intracellular probe fluorescence). The de-esterified form of CM-H_2_DCFDA showed a cytoplasmatic localization as indicated by the diffused intracellular fluorescence observed in the representative images of Figures 7A,B showing control cells and cells exposed to 50 μg/mL PET NP respectively. The fluorescence increase was dose-dependent in the PET NP concentration range tested with a linear increase in the range from 10 μg/mL to 50 μg/mL (Figure 7C). This result suggests that PET NPs exposure was associated with intracellular oxidative stress potentially initiating a cascade of toxicity pathways that could underly the altered cellular function observed.

## 4 Discussion

Research on the biological and health effects of NPs, particularly at the cellular level, has recently advanced significantly due to their widespread environmental contamination (Yee et al., 2021). However, knowledge gaps remain, limiting a full understanding of NPs effects on human health and ecosystems. Indeed, many studies focus on specific NP types, like polystyrene, which may not reflect the NP environmental diversity, reducing the generalizability of findings (Jayavel et al., 2024; Mahmud et al., 2024; Schröter and Ventura, 2022; Lehner et al., 2019). Additionally, research is often restricted to a few cell types.

The present work contributes to widening the knowledge of the cellular effects of NPs focusing on NIH-3T3 cells as fibroblast cell model and investigating the impact of exposure to PET NPs on key aspects of cell physiology including cell vitality, migration and redox balance. The cells were exposed to PET NPs obtained by a fragmentation process of PET close to the mechanical abrasion of plastic material occurring in the environment (Lionetto et al., 2022). Compared to previous works mainly based on round-shaped commercial PS NPs, the PET NPs used in this study offer several innovative aspects including their morphology and their intrinsic autofluorescence. They showed a polydisperse size distribution and irregular shapes and surface that closely mimic NPs found in the environment. Moreover, they exhibited intrinsic autofluorescence, eliminating the need for the conjugation with fluorescent dyes and in turn avoiding issues like dye leaching and potential toxicity introduced by the conjugated dyes. The PET NPs used in the present study were characterized by an intrinsic autofluorescence (Lionetto et al., 2022). When excited at 405 nm they expressed a maximum emission at 450 nm. This autofluorescence is attributed to intrinsic fluorescence properties of the polymer ascribable to the presence of aromatic groups in its chemical structure which can absorb photons at specific wavelengths and subsequently re-emit photons at a longer wavelength, generating autofluorescence (Allen et al., 2000; Lionetto et al., 2022). In this work the autofluorescence of PET NPs was assessed by confocal microscopy using a 405 nm laser line of A1 NIKON confocal laser scanning unit (emission filter 425–475 nm) coupled with a NIKON Ti microscope, demonstrating that the spectral properties of PET NPs make them suitable for spectrofluorimetry and fluorescence microscopy applications since they showed an excitation and emission peak compatible with the commercially available UV/violet light source and microscope’s filters and detectors.

We detected the internalization of PET NPs into 3T3 fibroblasts after 24 h exposure. The internalized PET NPs showed a cytoplasmatic distribution as can be assessed by merging brightfield and fluorescence images acquired by confocal microscopy. The internalization was dose-dependent. This result agrees with our previous data demonstrating the ability of these PET NPs to be internalized in another cell model represented by hemocytes of the bioindicator species *Mytilus galloprovincialis* under *in vitro* study (Lionetto et al., 2022). The internalization of NPs has been widely demonstrated for polystyrene NPs in several cell types, including rat basophilic leukemia cells (RBL-2H3) (Liu et al., 2021), human lung epithelial cells A549 (Xu et al., 2019), THP-1 cells from a human monocytic leukemia cell line (Liu et al., 2024), gastric epithelial (GES-1) cells (Ding et al., 2021), and human induced pluripotent stem cells (hiPSCs). It is known that polystyrene NPs can be internalized into cells through various pathways, including passive membrane transport and active endocytosis (Hua and Wang, 2022; Jeong et al., 2022) (Liu et al., 2021). Smaller particles (e.g., 50 nm) were internalized more efficiently than larger ones (e.g., 500 nm) (Liu et al., 2021) (Liu et al., 2024). The passive membrane penetration is due to the partition of polystyrene NPs in the water-phospholipid system thanks to hydrophobic interactions and Van der Waals’ forces as assessed on model membranes (Liu et al., 2021). On the other hand, NPs endocytosis pathways included clathrin-mediated, caveolin-mediated, and micropinocytosis (Liu et al., 2021; Ding et al., 2021) (Liu et al., 2024). Compared to the studies on polystyrene NPs, the available data on cellular internalization of PET NP is limited. To the best of our knowledge only a few studies investigated PET NPs cellular internalization. Magrì et al. (2018) demonstrated PET nanoparticle (NP) internalization into endolysosomes of Caco-2 intestinal cells at concentrations similar to the concentrations used in this study. Rodríguez-Hernández et al. (2019) and Aguilar-Guzmán et al. (2022) observed PET NP uptake in RAW macrophages, while Zhang et al. (2022) reported endocytotic uptake in A549 pulmonary cells after 24 h. In our experimental model we observed a cytoplasmatic distribution of PET NPs allowing to hypothesize an endocytotic pathway as a possible NPs uptake mechanism. However, the contribution of different internalization mechanisms cannot be excluded according to the heterogeneous dimensions and shape of the NPs used in the study. Indeed, both the size and shape of NPs significantly influence their internalization into cells (Liu et al., 2021; Agarwal et al., 2013) (Xu et al., 2019). Our results demonstrated for the first time fibroblasts were able to incorporate PET NPs within a short time period of exposure in a dose-dependent manner. However, based on the current results, we are not yet able to determine the precise subcellular localization of NPs. Future studies will be necessary to accurately identify the subcellular localization and to in deep characterize the internalization mechanisms of PET NPs into the cells, including a size-resolved analysis of the uptake.

Once assessed the internalization, we investigated potential cellular effects. PET NPs slightly but significantly decreased cell viability after 24 h exposure reaching the maximum effect of 20% viability inhibition at 50 μg/mL. This result agrees with data obtained by Zhang et al. (2022) on A549 cells using commercial PET NPs sized from 164 to 190 nm in a similar concentration range. However, we observed PET NPs able to interfere with an important cellular function in fibroblast physiology represented by cell migration and wound healing. The *in vitro* experimental model used to mimic aspects of wound repair was the so-called scratch test, which involves creating a scratch in the cell monolayer and observing and quantifying how cells migrate to close the gap. While the scratch test is valuable for studying cell migration, it does not fully replicate the complexity of *in vivo* wound healing, which involves multiple tissue layers, immune responses, and other physiological factors. Instead, the scratch test primarily evaluates cellular behaviours such as cell migration (how cells move to cover the scratch area) and cell proliferation (how cells proliferate to fill the wound area) (Grada et al., 2017; Suarez-Arnedo et al., 2020). About 60% reduction in the gap closure percentage of 3T3 cells was assessed by scratch test. Fibroblast migration is a complex, highly regulated process essential for wound healing, tissue repair, and development. This process involves coordinated events, including cytoskeletal reorganization, cell adhesion dynamics, and extracellular matrix (ECM) interactions (Tschumperlin, 2013). Obtained results suggest possible direct or indirect interference of PET NPs with the mechanisms underlying fibroblast migration and their involvement in wound healing. Alterations in cell migration induced by NPs exposure have been previously demonstrated in other cell types and the effects appeared cell type and NP specific. Polystyrene NPs inhibited the migration and invasion of human trophoblast cells by disrupting the ROCK1 pathway, triggering autophagy, and degrading the key transcription factor SOX2 (Wan et al., 2024). In astrocytes derived from neural stem cells, both polystyrene nano- and microplastics modify the expression of genes involved in cell migration, suggesting that these particles can affect cellular motility (Marcellus et al., 2024). In addition, polyethylene NPs enhanced the migration potential of the cells through mechanisms involving Epithelial–Mesenchymal Transition and, in turn, raising concern for possible role in carcinogenesis (Traversa et al., 2024).

Intracellular reactive oxygen species (ROS) are considered key regulators of cell motility (Huang et al., 2013). They are known to exert a dual role, promoting migration at low concentrations but inhibiting cell motility when excessively produced, disrupting cytoskeletal integrity, adhesion turnover, and mitochondrial function (Huang et al., 2013; Dunagan and Rao, 2009). The ROS effects on cell motility are highly context-dependent, influenced by the type of cells and the specific oxidative stress conditions (Huang et al., 2013; Dunagan and Rao, 2009) (P. Xu et al., 2020). In fibroblasts, it has been demonstrated that modulating reactive oxygen species (ROS) levels plays a crucial role in regulating wound healing (Janda et al., 2016) and that excess intracellular ROS can impair fibroblast migration (Fujiwara et al., 2019; Loo and Halliwell, 2012). In our experimental model, using the cell permeant dichlorofluorescein diacetate probe (CM-H_2_DCFDA), we demonstrated that PET NPs significantly increased the ROS intracellular contents, which in turn suggested the induction of an oxidative stress condition in 3T3 cells. The effect was dose-dependent with a linear increase in the concentration range 10–50 μg/mL and a saturating behaviour at the highest concentration tested of 100 μg/mL. The saturating behavior could be ascribed to the fact that the cellular uptake of NPs may be limited by the endocytic capacity of the cell. Previous studies on other cellular models demonstrated that endocytosis of nanoparticles shows saturation with increased concentration and time (Sun et al., 2022). Once this capacity is saturated, it is possible that no further NPs are internalized and no further increase in cellular effects can be observed. Moreover, the exocytosis of internalized PET NPs should also be considered in this process and could contribute to determining the observed plateau. Recent studies on polystyrene NPs have documented NPs exocytosis in different cell types (Liu et al., 2023; Han and Ryu, 2022). Further research is required to address this aspect.

The marked dose-response ROS increase observed in our experimental cell model suggests that oxidative stress induction could represent an early step in the cascade of events induced by PET NPs exposure earlier to more integrated endpoints. The effects detected on 3T3 fibroblast confirm evidence obtained from other cell types such as primary human nasal epithelial cells (Annangi et al., 2023), where an increase of the intracellular ROS concentration following PET NPs exposure was associated to the alteration of mitochondrial membrane functionality. Increases ROS production and consequent oxidative stress induced by PET NPs have also been detected in *Saccharomyces cerevisiae* (Kaluç et al., 2024), and primary nasal epithelial cells (HNEpCs) (Annangi et al., 2023). As regards possible mechanisms to explain the PET induced intracellular increase of ROS concentration and in turn oxidative stress in 3T3 fibroblasts, we can hypothesize possible alteration in the mitochondrial functions since mitochondrial respiratory chain is a primary source of intracellular ROS. It is known that dysfunction in mitochondria leads to increased ROS production (Murphy, 2013). Mitochondria can respond in turn to elevated ROS levels by further increasing their own ROS production, a process known as ROS-induced ROS release (Brady et al., 2006). Moreover, PET NPs induced alterations in the antioxidant defence of the cells could be another possible mechanism contributing to the enhanced ROS concentration in our experimental model. It is known that NPs can significantly alter the activity of antioxidant enzymes across various cell types and organisms (Z. Liu et al., 2020; Babaei et al., 2022; Polo et al., 2024). Although most of the information available on this aspect arises from studies on polystyrene NPs, these effects could also be involved in the PET NPs oxidative stress induction.

Overall, the integrated analysis of scratch test and ROS data suggests that PET NPs induced ROS production could interfere with cell migration in 3T3 cells, possibly representing an underlying early toxicity mechanism. Although future studies will be addressed to clarify this issue, the present work for the first time demonstrated PET NPs able to impair cell migration in fibroblasts with implication in wound healing in association with induced ROS increase.

## 5 Conclusion

This study analyzes the effects of PET NPs on fibroblasts cells using the NIH-3T3 murine model. The PET NPs used, produced through a top-down approach, closely resemble environmental NPs and exhibit intrinsic autofluorescence, facilitating NP interaction studies without the need for additional fluorescent labels. Furthermore, the determination of the Hansen solubility parameters of the PET NPs and culture medium provides valuable insights into PET NPs interaction behavior, contributing to a deeper understanding of NPs behavior in biological systems.

PET NPs can be internalized by fibroblasts and localize in the cytoplasm in a dose-dependent manner. They induce a slightly dose-dependent decrease in cell viability within 20% inhibition in the concentration range used (10–100 μg/mL) but a more marked oxidative stress due to increase intracellular ROS concentration which could be a key mechanism of toxic effect inside the cell. PET NP exposure impaired fibroblast migration, as observed in a wound healing assay suggesting potential interference with tissue repair and wound healing. Overall, this study underscores the potential hazards of PET NPs on fibroblasts, which play a crucial role in wound healing and tissue maintenance. The observed oxidative stress and impaired migration suggest that PET NPs may exert negative effect on fibroblasts, warranting further investigation into their interference and underlying mechanisms.

## Data Availability

The raw data supporting the conclusions of this article will be made available by the authors, without undue reservation.

## References

[B1] Abafe O. A. Harrad S. Abdallah M. A.-E. (2023). Novel insights into the dermal bioaccessibility and human exposure to brominated flame retardant additives in microplastics. Environ. Sci. and Technol. 57 (29), 10554–10562. 10.1021/acs.est.3c01894 37450894 PMC10373483

[B2] Agarwal R. Singh V. Jurney P. Shi L. Sreenivasan S. V. Roy K. (2013). Mammalian cells preferentially internalize hydrogel nanodiscs over nanorods and use shape-specific uptake mechanisms. Proc. Natl. Acad. Sci. U. S. A. 110 (43), 17247–17252. 10.1073/pnas.1305000110 24101456 PMC3808581

[B3] Aguilar-Guzmán J. C. Bejtka K. Fontana M. Valsami-Jones E. Villezcas A. M. Vazquez-Duhalt R. (2022). Polyethylene terephthalate nanoparticles effect on RAW 264.7 macrophage cells. Microplast. Nanoplast. 2 (1), 9. 10.1186/s43591-022-00027-1

[B76] Allen N. S. Rivalle G. Edge M. Roberts I. Fagerburg D. R. (2000). Characterisation and identification of fluorescent hydroxylated terephthalate species in the thermal and UV degradation of poly (ethylene-co-1, 4-cyclohexanedimethylene terephthalate)(PECT). Polym. Degrad. Stab. 67, 325–334.

[B4] Annangi B. Villacorta A. Vela L. Tavakolpournegari A. Marcos R. Hernández A. (2023). Effects of true-to-life PET nanoplastics using primary human nasal epithelial cells. Environ. Toxicol. Pharmacol. 100, 104140. 10.1016/j.etap.2023.104140 37137422

[B5] Babaei A. A. Rafiee M. Khodagholi F. Ahmadpour E. Amereh F. (2022). Nanoplastics-induced oxidative stress, antioxidant defense, and physiological response in exposed Wistar albino rats. Environ. Sci. Pollut. Res. 29 (8), 11332–11344. 10.1007/s11356-021-15920-0 34535860

[B6] Bidashimwa D. Hoke T. Huynh T. B. Narkpitaks N. Priyonugroho K. Ha T. T. (2023). Plastic pollution: how can the global health community fight the growing problem? In BMJ Glob. Health 8. e012140. 10.1136/bmjgh-2023-012140 PMC10277055 37295791

[B7] Boncler M. Lukasiak M. Dastych J. Golanski J. Cezary W. (2018). Differentiated mitochondrial function in mouse 3T3 fibroblasts and human epithelial or endothelial cells in response to chemical exposure. Basic Clin. Pharmacol. Toxicol. 124, 199–210. 10.1111/bcpt.13117 30137675

[B8] Brady N. R. Hamacher-Brady A. Westerhoff H. V. Gottlieb R. A. (2006). A wave of reactive oxygen species (ROS)-Induced ROS release in a Sea of excitable mitochondria. Https//Home.Liebertpub.Com/Ars 8 (9–10), 1651–1665. 10.1089/ARS.2006.8.1651 16987019

[B9] Caputi S. Diomede F. Lanuti P. Marconi G. D. Di Carlo P. Sinjari B. (2022). Microplastics affect the inflammation pathway in human gingival fibroblasts: a study in the Adriatic Sea. Int. J. Environ. Res. Public Health 19 (13), 7782. 10.3390/ijerph19137782 35805437 PMC9266176

[B10] Celebi Sözener Z. Cevhertas L. Nadeau K. Akdis M. Akdis C. A. (2020). Environmental factors in epithelial barrier dysfunction. J. Allergy Clin. Immunol. 145 (6), 1517–1528. 10.1016/j.jaci.2020.04.024 32507229

[B11] Ding Y. Zhang R. Li B. Du Y. Li J. Tong X. (2021). Tissue distribution of polystyrene nanoplastics in mice and their entry, transport, and cytotoxicity to GES-1 cells. Environ. Pollut. 280, 116974. 10.1016/j.envpol.2021.116974 33784569

[B12] Dube E. Okuthe G. E. (2023). Plastics and micro/nano-plastics (MNPs) in the environment: occurrence, impact, and toxicity. Int. J. Environ. Res. Public Health 20 (17), 6667. 10.3390/ijerph20176667 37681807 PMC10488176

[B13] Dunagan M. Rao R. K. (2009). Oxidative stress attenuates epithelial differentiation and accelerates cell migration in caco-2 cells. FASEB J. 23 (S1), 979.1. 10.1096/FASEBJ.23.1_SUPPLEMENT.979.1

[B77] Ferrante M. C. Monnolo A. Del Piano F. Mattace Raso G. Meli R. (2022). The pressing issue of micro- and nanoplastic contamination: profiling the reproductive alterations mediated by oxidative stress. Antioxidants 11 (2), 193. 10.3390/antiox11020193 35204076 PMC8868557

[B14] Ferreira R. O. G. Nag R. Gowen A. Xu J. L. (2024). Deciphering the cytotoxicity of micro- and nanoplastics in Caco-2 cells through meta-analysis and machine learning. Environ. Pollut. 362, 124971. 10.1016/j.envpol.2024.124971 39293654

[B15] Forman H. J. Augusto O. Brigelius-Flohe R. Dennery P. A. Kalyanaraman B. Ischiropoulos H. (2015). Even free radicals should follow some rules: a Guide to free radical research terminology and methodology. Free Radic. Biol. Med. 78, 233–235. 10.1016/J.FREERADBIOMED.2014.10.504 25462642

[B16] Fujiwara T. Dohi T. Maan Z. N. Rustad K. C. Kwon S. H. Padmanabhan J. (2019). Age-associated intracellular superoxide dismutase deficiency potentiates dermal fibroblast dysfunction during wound healing. Exp. Dermatol. 28 (4), 485–492. 10.1111/EXD.13404 28677217

[B78] Gettings S. M. Timbury W. Dmochowska A. Sharma R. McGonigle R. MacKenzie L. E. (2024). Polyethylene terephthalate (PET) micro- and nanoplastic particles affect the mitochondrial efficiency of human brain vascular pericytes without inducing oxidative stress. NanoImpact. 34, 100508. 10.1016/j.impact.2024.100508 38663501

[B85] Giannakopoulos E. Katopodi A. Rallis M. Politopoulos K. Alexandratou E. (2023). The effects of low power laser light at 661 nm on wound healing in a scratch assay fibroblast model. Lasers Med. Sci. 38 (27), 27. 10.1007/s10103-022-03670-5 PMC9794538 36574084

[B17] Gigault J. Halle A. T. Baudrimont M. Pascal P. Y. Gauffre F. Phi T. L. (2018). Current opinion: what is a nanoplastic? Environ. Pollut. 235, 1030–1034. 10.1016/j.envpol.2018.01.024 29370948

[B18] Giordano M. E. Caricato R. Lionetto M. G. (2020a). Concentration dependence of the antioxidant and prooxidant activity of trolox in hela cells: involvement in the induction of apoptotic volume decrease. Antioxidants 9 (11), 1058–1112. 10.3390/antiox9111058 33137938 PMC7693461

[B19] Giordano M. E. Caricato R. Verri T. Lionetto M. G. (2020b). The colon epithelium as a target for the intracellular antioxidant activity of hydroxytyrosol: a study on rat colon explants. J. Funct. Foods 64, 103604. 10.1016/j.jff.2019.103604

[B20] Giordano M. E. Lionetto M. G. (2023). Intracellular redox behavior of quercetin and resveratrol singly and in mixtures. Molecules 28 (12), 4682. 10.3390/MOLECULES28124682 37375237 PMC10301869

[B21] Giordano M. E. Udayan G. Guascito M. R. De Bartolomeo A. R. Carlino A. Conte M. (2023). Apoptotic volume decrease (AVD) in A549 cells exposed to water-soluble fraction of particulate matter (PM10). Front. Physiol. 14, 1218687. 10.3389/fphys.2023.1218687 37492639 PMC10364053

[B22] Grada A. Otero-Vinas M. Prieto-Castrillo F. Obagi Z. Falanga V. (2017). Research techniques made simple: analysis of collective cell migration using the wound healing assay. J. Invest. Dermatol. 137 (2), e11–e16. 10.1016/j.jid.2016.11.020 28110712

[B23] Greco A. Lionetto F. Maffezzoli A. (2015). Processing and characterization of amorphous polyethylene terephthalate fibers for the alignment of carbon nanofillers in thermosetting resins. Polym. Compos. 36 (6), 1096–1103. 10.1002/PC.23366

[B24] Han S. W. Ryu K. Y. (2022). Increased clearance of non-biodegradable polystyrene nanoplastics by exocytosis through inhibition of retrograde intracellular transport. J. Hazard. Mater. 439, 129576. 10.1016/j.jhazmat.2022.129576 35850071

[B25] Hansen C. M. (2007). Hansen solubility parameters a User’s Handbook. Second Edition.

[B26] Hua X. Wang D. (2022). Cellular uptake, transport, and organelle response after exposure to microplastics and nanoplastics: current knowledge and perspectives for environmental and health risks. Cell. Uptake, Transp. Organelle Response After Expo. Microplast. Nanoplast. Curr. Knowl. Perspect. Environ. Health Risks 260, 12. 10.1007/s44169-022-00013-x

[B27] Huang J.-S. Cho C.-Y. Hong C.-C. Yan M.-D. Hsieh M.-C. Lay J.-D. (2013). Oxidative stress enhances Axl-mediated cell migration through an Akt1/Rac1-dependent mechanism. Free Radic. Biol. Med. 65, 1246–1256. 10.1016/j.freeradbiomed.2013.09.011 24064382

[B28] Janda J. Nfonsam V. Calienes F. Sligh J. E. Jandova J. (2016). Modulation of ROS levels in fibroblasts by altering mitochondria regulates the process of wound healing. Arch. Dermatol. Res. 308 (4), 239–248. 10.1007/s00403-016-1628-9 26873374

[B29] Jayavel S. Govindaraju B. Michael J. R. Viswanathan B. (2024). Impacts of micro and nanoplastics on human health. Bull. Natl. Res. Centre 48 (1), 110. 10.1186/s42269-024-01268-1

[B30] Jeong H. Kim W. Choi D. Heo J. Han U. Jung S. Y. (2022). Potential threats of nanoplastic accumulation in human induced pluripotent stem cells. Chem. Eng. J. 427, 131841. 10.1016/j.cej.2021.131841

[B31] Kaluç N. Çötelli E. L. Tuncay S. Thomas P. B. (2024). Polyethylene terephthalate nanoplastics cause oxidative stress induced cell death in *Saccharomyces cerevisiae* . J. Environ. Sci. Health, Part A 59 (4), 180–188. 10.1080/10934529.2024.2345026 38693670

[B32] Kendall R. T. Feghali-Bostwick C. A. Murray L. A. Ltd M. Seta F. (2014). Fibroblasts in fibrosis: novel roles and mediators. Front. Pharmacol. 5, 123. 10.3389/fphar.2014.00123 24904424 PMC4034148

[B33] Khan A. Jia Z. (2023). Recent insights into uptake, toxicity, and molecular targets of microplastics and nanoplastics relevant to human health impacts. iScience. 10.1016/j.isci.2023.106061 PMC9929686 36818296

[B34] King B. A. Oh D. H. (2004). Spatial control of reactive oxygen species formation in fibroblasts using two-photon excitation. Photochem. Photobiol. 80, 1–6. 10.1562/2004-03-01-RA-093.1 15339206 PMC2774523

[B35] Larese Filon F. Bello D. Cherrie J. W. Sleeuwenhoek A. Spaan S. Brouwer D. H. (2016). Occupational dermal exposure to nanoparticles and nano-enabled products: Part I—factors affecting skin absorption. Int. J. Hyg. Environ. Health 219 (6), 536–544. 10.1016/j.ijheh.2016.05.009 27289581

[B36] Lehner R. Weder C. Petri-Fink A. Rothen-Rutishauser B. (2019). Emergence of nanoplastic in the environment and possible impact on human health. Environ. Sci. and Technol. 53 (4), 1748–1765. 10.1021/acs.est.8b05512 30629421

[B79] Leslie H. A. van Velzen M. J. M. Brandsma S. H. Vethaak A. D. Garcia-Vallejo J. J. Lamoree M. H. (2022). Discovery and quantification of plastic particle pollution in human blood. Environ. Int. 163, 107199. 10.1016/j.envint.2022.107199 35367073

[B37] Lionetto F. Esposito Corcione C. Messa F. Perrone S. Salomone A. Maffezzoli A. (2023). The sorption of amoxicillin on engineered polyethylene terephthalate microplastics. J. Polym. Environ. 31, 1383–1397. 10.1007/s10924-022-02690-0

[B38] Lionetto F. Esposito Corcione C. Rizzo A. Maffezzoli A. Ramanavicius A. (2021). Production and characterization of polyethylene terephthalate nanoparticles. Polym. (Basel). 13, 3745. 10.3390/polym13213745 PMC8587476 34771306

[B39] Lionetto F. Lionetto M. G. Mele C. Corcione C. E. Bagheri S. Udayan G. (2022). Autofluorescence of model polyethylene terephthalate nanoplastics for cell interaction studies. Nanomaterials 12 (9), 1560. 10.3390/nano12091560 35564269 PMC9100011

[B40] Liu L. Xu K. Zhang B. Ye Y. Zhang Q. Jiang W. (2021). Cellular internalization and release of polystyrene microplastics and nanoplastics. Sci. Total Environ. 779, 146523. 10.1016/j.scitotenv.2021.146523 34030247

[B41] Liu Y. Y. Liu J. Wu H. Zhang Q. Tang X. R. Li D. (2023). Endocytosis, distribution, and exocytosis of polystyrene nanoparticles in human lung cells. Nanomaterials 13 (1), 84. 10.3390/NANO13010084/S1 PMC9824409 36615994

[B42] Liu Z. Huang Y. Jiao Y. Chen Q. Wu D. Yu P. (2020). Polystyrene nanoplastic induces ROS production and affects the MAPK-HIF-1/NFkB-mediated antioxidant system in *Daphnia pulex* . Aquat. Toxicol. 220, 105420. 10.1016/j.aquatox.2020.105420 31986404

[B43] Liu Z. Wang G. Sheng C. Zheng Y. Tang D. Zhang Y. (2024). Intracellular protein adsorption behavior and biological effects of polystyrene nanoplastics in THP-1 cells. Environ. Sci. and Technol. 58 (6), 2652–2661. 10.1021/acs.est.3c05493 38294362

[B44] Loo A. E. K. Halliwell B. (2012). Effects of hydrogen peroxide in a keratinocyte-fibroblast co-culture model of wound healing. Biochem. Biophys. Res. Commun. 423 (2), 253–258. 10.1016/j.bbrc.2012.05.100 22634311

[B45] Magrì D. Sánchez-Moreno P. Caputo G. Gatto F. Veronesi M. Bardi G. (2018). Laser ablation as a versatile Tool to mimic polyethylene terephthalate nanoplastic pollutants: characterization and toxicology assessment. ACS Nano 12 (8), 7690–7700. 10.1021/acsnano.8b01331 29944342

[B46] Mahmud F. Sarker D. B. Jocelyn J. A. Sang Q. X. A. (2024). Molecular and cellular effects of microplastics and nanoplastics: focus on inflammation and senescence. Cells 13 (21), 1788. 10.3390/cells13211788 39513895 PMC11545702

[B47] Marcellus K. A. Bugiel S. Nunnikhoven A. Curran I. Gill S. S. (2024). Polystyrene nano- and microplastic particles induce an inflammatory gene expression profile in rat neural stem cell-derived astrocytes *in vitro* . Nanomaterials 14 (5), 429. 10.3390/nano14050429 38470760 PMC10935329

[B48] Murphy M. P. (2013). Mitochondrial dysfunction indirectly elevates ROS production by the endoplasmic reticulum. Cell Metab. 18 (2), 145–146. 10.1016/j.cmet.2013.07.006 23931748

[B49] Nihart A. J. Garcia M. A. El Hayek E. Liu R. Olewine M. Kingston J. D. (2025). Bioaccumulation of microplastics in decedent human brains. Nat. Med. 31, 1114–1119. 10.1038/s41591-024-03453-1 39901044 PMC12003191

[B50] Ollivier V. Chabbat J. Herbert J. M. Hakim J. De Prost D. (2000). Vascular endothelial growth factor production by fibroblasts in response to factor VIIa binding to tissue factor involves thrombin and factor Xa. Arterioscler., Thromb., Vasc. Biol. 20 (5), 1374–1381. 10.1161/01.atv.20.5.1374 10807756

[B51] Peng M. Félix R. C. Canário A. V. M. Power D. M. (2024). The physiological effect of polystyrene nanoplastic particles on fish and human fibroblasts. Sci. Total Environ. 914, 169979. 10.1016/j.scitotenv.2024.169979 38215851

[B52] Plikus M. V. Wang X. Sinha S. Forte E. Thompson S. M. Herzog E. L. (2021). Fibroblasts: origins, definitions, and functions in health and disease. Cell 184 (15), 3852–3872. 10.1016/j.cell.2021.06.024 34297930 PMC8566693

[B53] Polo G. Lionetto F. Giordano M. E. Lionetto M. G. (2024). Interaction of micro- and nanoplastics with enzymes: the case of carbonic anhydrase. Int. J. Mol. Sci. 25 (17), 9716. 10.3390/ijms25179716 39273668 PMC11396312

[B54] Rahimi A. M. Cai M. Hoyer-Fender S. (2022). Heterogeneity of the NIH3T3 fibroblast cell line. Cells 11, 2677. 10.3390/cells11172677 36078083 PMC9455036

[B55] Rajabimashhadi Z. Gallo N. Russo F. Ghiyami S. Mele C. Giordano M. E. (2024). Production and physico-chemical characterization of nano-sized collagen from equine tendon. Int. J. Biol. Macromol. 277, 134220. 10.1016/j.ijbiomac.2024.134220 39069054

[B56] Rodríguez-Hernández A. G. Muñoz-Tabares J. A. Aguilar-Guzmán J. C. Vazquez-Duhalt R. (2019). A novel and simple method for polyethylene terephthalate (PET) nanoparticle production. Environ. Sci. Nano 6 (7), 2031–2036. 10.1039/C9EN00365G

[B57] Sadeghinia H. Hanachi P. Ramezani R. Karbalaei S. (2025). Toxic effects of polystyrene nanoplastics on MDA-MB-231 breast cancer and HFF-2 normal fibroblast cells: viability, cell death, cell cycle and antioxidant enzyme activity. Environ. Sci. Eur. 37, 1. 10.1186/s12302-024-01026-0

[B58] Schröter L. Ventura N. (2022). Nanoplastic toxicity: insights and challenges from experimental model systems. Small 18 (31), e2201680. 10.1002/SMLL.202201680 35810458

[B59] Silzle T. Randolph G. J. Kreutz M. Kunz-Schughart L. A. (2003). The fibroblast: sentinel cell and local immune modulator in tumor tissue. Int. J. Cancer 108, 173–180. 10.1002/ijc.11542 14639599

[B60] Stojkovic M. Ortuño Guzmán F. M. Han D. Stojkovic P. Dopazo J. Stankovic K. M. (2023). Polystyrene nanoplastics affect transcriptomic and epigenomic signatures of human fibroblasts and derived induced pluripotent stem cells: implications for human health. Environ. Pollut. (Barking, Essex) 1987, 120849. 10.1016/J.ENVPOL.2022.120849 36509347

[B61] Suarez-Arnedo A. Torres F. Id F. Clavijo C. Arbelá Ez P. Cruz J. C. (2020). An image J plugin for the high throughput image analysis of *in vitro* scratch wound healing assays. PLoS One 15, e0232565. 10.1371/journal.pone.0232565 32722676 PMC7386569

[B62] Sun W. Tian Y. Wang Z. Zhang H. Zheng A. (2022). The study of cyclosporin A nanocrystals uptake and transport across an intestinal epithelial cell model. Polymers 14 (10), 1975. 10.3390/POLYM14101975 35631858 PMC9147483

[B63] Swee-Li Yee M. Hii L.-W. King Looi C. Lim W.-M. Wong S.-F. Kok Y.-Y. (2021). Impact of microplastics and nanoplastics on human health. Nanomater. (Basel). 11, 496. 10.3390/nano11020496 PMC7920297 33669327

[B64] Sylvester P. W. (2011). “Optimization of the tetrazolium dye (MTT) colorimetric assay for cellular growth and viability,” in Drug design and discovery: methods and protocols. Editor Satyanarayanajois S. D. (Totowa, NJ: Humana Press), 157–168. 10.1007/978-1-61779-012-6_9 21318905

[B65] Tracy L. E. Minasian R. A. Caterson E. J. (2016). Extracellular matrix and dermal fibroblast function in the healing wound. Adv. Wound Care 5 (3), 119–136. 10.1089/WOUND.2014.0561 PMC4779293 26989578

[B66] Traversa A. Mari E. Pontecorvi P. Gerini G. Romano E. Megiorni F. (2024). Polyethylene micro/nanoplastics exposure induces epithelial–mesenchymal transition in human bronchial and alveolar epithelial cells. Int. J. Mol. Sci. 25 (18), 10168. 10.3390/ijms251810168 39337653 PMC11432389

[B67] Tschumperlin D. J. (2013). Fibroblasts and the ground they walk on. Physiology 28 (6), 380–390. 10.1152/physiol.00024.2013 24186933 PMC3858213

[B68] Wagner G. Sievers L. Tiburcy M. Zimmermann W. H. Kollmar O. Schmalz G. (2022). Impact of immunosuppressive drugs on fibroblasts: an *in vitro* study. J. Clin. Med. 11, 3107. 10.3390/jcm11113107 35683494 PMC9181118

[B69] Wan S. Wang X. Chen W. Xu Z. Zhao J. Huang W. (2024). Polystyrene nanoplastics activate autophagy and suppress trophoblast cell migration/invasion and migrasome formation to induce miscarriage. ACS Nano 18 (4), 3733–3751. 10.1021/acsnano.3c11734 38252510

[B80] Wu Q. Liu C. Liu D. Wang Y. Qi H. Liu X. (2024). Polystyrene nanoplastics-induced lung apoptosis and ferroptosis via ROS-dependent endoplasmic reticulum stress. Sci. Total Environ. 912, 169260. 10.1016/j.scitotenv.2023.169260 38086481

[B70] Xu M. Halimu G. Zhang Q. Song Y. Fu X. Li Y. (2019). Internalization and toxicity: a preliminary study of effects of nanoplastic particles on human lung epithelial cell. Sci. Total Environ. 694, 133794. 10.1016/j.scitotenv.2019.133794 31756791

[B71] Xu P. Xue Y. N. Ji H. H. Tan C. Guo S. (2020). H2O2-induced oxidative stress disrupts mitochondrial functions and impairs migratory potential of human epidermal melanocytes. Exp. Dermatol. 29 (8), 733–741. 10.1111/EXD.14134 32580253

[B72] Yadav D. Savjani J. Savjani K. Shah H. (2023). Exploring potential coformer screening techniques based on experimental and virtual strategies in the manufacturing of pharmaceutical cocrystal of efavirenz. J. Pharm. Innovation 18 (3), 1128–1144. 10.1007/S12247-022-09704-3/TABLES/6

[B73] Yee M. S. L. Hii L. W. Looi C. K. Lim W. M. Wong S. F. Kok Y. Y. (2021). Impact of microplastics and nanoplastics on human health. Nanomaterials 11 (2), 496. 10.3390/NANO11020496 33669327 PMC7920297

[B74] Zarus G. M. Muianga C. Hunter C. M. Pappas R. S. (2021). A review of data for quantifying human exposures to micro and nanoplastics and potential health risks. Sci. Total Environ. 756, 144010. 10.1016/j.scitotenv.2020.144010 33310215 PMC7775266

[B75] Zhang H. Zhang S. Duan Z. Wang L. (2022). Pulmonary toxicology assessment of polyethylene terephthalate nanoplastic particles *in vitro* . Environ. Int. 162, 107177. 10.1016/j.envint.2022.107177 35303532

