# Supplementary material for: Impact of polyethylene terephthalate nanoplastics (PET) on fibroblasts: a study on NIH-3T3 cells
Source: Front Physiol. 2025 Jun 9;16:1580682. doi: 10.3389/fphys.2025.1580682 (PMC12183297; doi:10.3389/fphys.2025.1580682)

Figure S1: Representative image (of n=3 independent experiments) of 3T3 fibroblasts exposed to PET NPs (50 µg/ml) for 24h visualized in brightfield (C) and confocal microscopy (D) by a 405 nm laser line of A1 NIKON confocal laser scanning unit coupled with a NIKON Ti microscope. Control cells (A,B) are shown for comparison. Objective used 60X oil immersion.

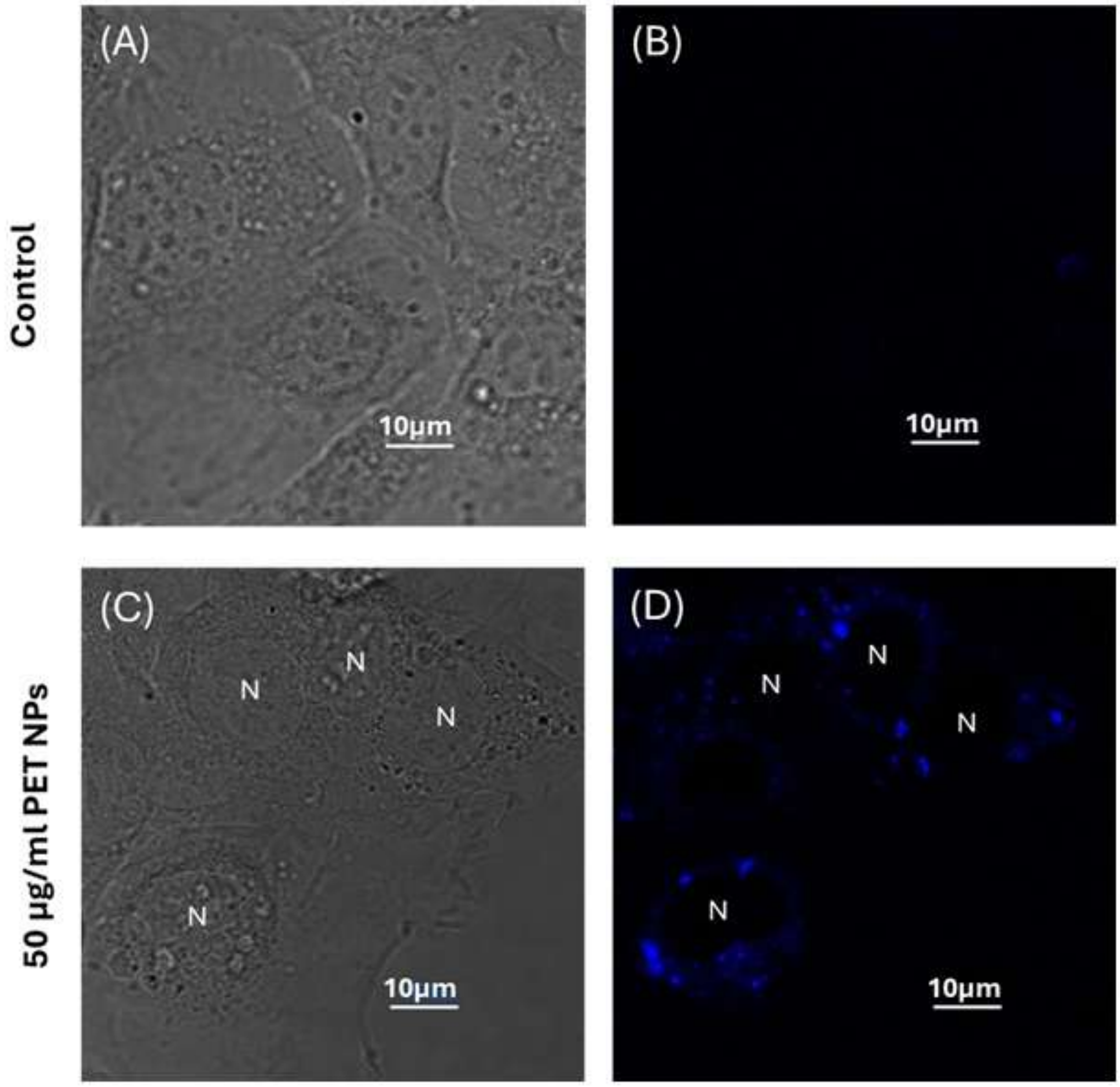

Supplement: Supplementary file 1 [file DataSheet1.pdf]
